# Clarithromycin Inhibits Pneumolysin Production via Downregulation of *ply* Gene Transcription despite Autolysis Activation

**DOI:** 10.1128/Spectrum.00318-21

**Published:** 2021-09-01

**Authors:** Hisanori Domon, Toshihito Isono, Takumi Hiyoshi, Hikaru Tamura, Karin Sasagawa, Tomoki Maekawa, Satoru Hirayama, Katsunori Yanagihara, Yutaka Terao

**Affiliations:** a Division of Microbiology and Infectious Diseases, Niigata University Graduate School of Medical and Dental Sciences, Niigata, Japan; b Center for Advanced Oral Science, Niigata University Graduate School of Medical and Dental Sciences, Niigata, Japan; c Division of Periodontology, Niigata University Graduate School of Medical and Dental Sciences, Niigata, Japan; d Department of Laboratory Medicine, Nagasaki University Graduate School of Biomedical Sciences, Nagasaki, Japan; Emory University School of Medicine

**Keywords:** *Streptococcus pneumoniae*, autolysis, clarithromycin, erythromycin, lung injury, macrolides, neutrophil elastase, pneumolysin, pneumonia

## Abstract

Streptococcus pneumoniae, the most common cause of community-acquired pneumonia, causes severe invasive infections, including meningitis and bacteremia. The widespread use of macrolides has been reported to increase the prevalence of macrolide-resistant S. pneumoniae (MRSP), thereby leading to treatment failure in patients with pneumococcal pneumonia. However, previous studies have demonstrated that several macrolides and lincosamides have beneficial effects on MRSP infection since they inhibit the production and release of pneumolysin, a pneumococcal pore-forming toxin released during autolysis. In this regard, we previously demonstrated that the mechanisms underlying the inhibition of pneumolysin release by erythromycin involved both the transcriptional downregulation of the gene encoding pneumolysin and the impairment of autolysis in MRSP. Here, using a cell supernatant of the culture, we have shown that clarithromycin inhibits pneumolysin release in MRSP. However, contrary to previous observations in erythromycin-treated MRSP, clarithromycin upregulated the transcription of the pneumococcal autolysis-related *lytA* gene and enhanced autolysis, leading to the leakage of pneumococcal DNA. On the other hand, compared to erythromycin, clarithromycin significantly downregulated the gene encoding pneumolysin. In a mouse model of MRSP pneumonia, the administration of both clarithromycin and erythromycin significantly decreased the pneumolysin protein level in bronchoalveolar lavage fluid and improved lung injury and arterial oxygen saturation without affecting bacterial load. Collectively, these *in vitro* and *in vivo* data reinforce the benefits of macrolides on the clinical outcomes of patients with pneumococcal pneumonia.

**IMPORTANCE** Pneumolysin is a potent intracellular toxin possessing multiple functions that augment pneumococcal virulence. For over 10 years, sub-MICs of macrolides, including clarithromycin, have been recognized to decrease pneumolysin production and release from pneumococcal cells. However, this study indicates that macrolides significantly slowed pneumococcal growth, which may be related to decreased pneumolysin release recorded by previous studies. In this study, we demonstrated that clarithromycin decreases pneumolysin production through downregulation of *ply* gene transcription, regardless of its inhibitory activity against bacterial growth. Additionally, administration of clarithromycin resulted in the amelioration of lung injury in a mouse model of pneumonia induced by macrolide-resistant pneumococci. Therefore, therapeutic targeting of pneumolysin offers a good strategy to treat pneumococcal pneumonia.

## INTRODUCTION

Streptococcus pneumoniae, a pneumococcus, is a Gram-positive Diplococcus and a commensal of the human nasopharynx. This bacterium commonly causes community-acquired pneumonia (CAP) and otitis media. In addition to these localized infections, pneumococcus also causes severe invasive infections, including meningitis and bacteremia, which continue to be a major cause of morbidity and mortality worldwide, especially in children under 5 years of age (1). A major concern for the treatment of these pneumococcal diseases is the increasing resistance of S. pneumoniae to most of the commonly prescribed antibiotics, including penicillin, macrolides, and to a lesser extent, fluoroquinolones (2). Our previous study showed that among the 2,415 pneumococcal clinical isolates in Japan between 2014 and 2017, 38, 82, and 0.1% of the isolates were nonsusceptible to benzylpenicillin, azithromycin, and levofloxacin, respectively (3).

The high prevalence of macrolide-resistant S. pneumoniae (MRSP) has led to the general consideration that organisms presenting with a high degree of macrolide resistance cannot be effectively treated with macrolide monotherapy (4). Although the 2007 clinical guideline of the American Thoracic Society strongly recommends macrolide monotherapy for outpatients with CAP, the 2019 guideline recommends the use of amoxicillin or doxycycline for empirical treatment and macrolides for conditional therapy only in areas where the pneumococcal resistance to macrolides is less than 25% (5). Additionally, several studies have suggested that *in vitro* macrolide resistance is associated with clinical treatment failure during severe pneumococcal infections (6, 7). In contrast, macrolide monotherapy has been reported to be an effective treatment option for adults with MRSP-induced CAP (8, 9). Cilloniz et al. reported that infection by MRSP did not worsen the clinical outcomes in CAP patients compared to infection by macrolide-sensitive strains (10). Additionally, treatment with macrolides has been associated with decreased mortality in patients with severe sepsis caused by MRSP (11). Therefore, although there is a contradiction between *in vitro* macrolide resistance and clinical outcomes, macrolides may have some beneficial effects on MRSP infection (4).

Several molecular mechanisms have been proposed to account for treatment success using macrolides. Reportedly, macrolides have immunomodulatory effects, that is, they decrease the concentration of proinflammatory cytokines and inhibit the release of superoxide anions by neutrophils (12). These effects are thought to ameliorate pulmonary function and airway infections. Furthermore, macrolides reduce pneumococcal pathogenicity by inhibiting the production and release of the pneumococcal cytotoxin, pneumolysin (PLY) (13, 14).

PLY is a cholesterol-dependent cytolysin that forms ring-like pores in host cell membranes and induces cell death, thereby augmenting pneumococcal virulence (15). A previous study reported that the PLY-negative mutants of S. pneumoniae show a significant reduction in virulence in mouse models of both pneumonia and intraperitoneal infections (16). Additionally, PLY can trigger proinflammatory responses through Toll-like receptor 4 (TLR 4) (17). However, S. pneumoniae does not actively secrete PLY because it lacks the signal sequences associated with the N terminus of the protein (18). One of the mechanisms underlying PLY release involves cellular autolysis, facilitated principally by the major autolysin, LytA. In this regard, we previously demonstrated that, compared to the sub-MICs of azithromycin (15-membered macrolide), those of erythromycin (ERY; 14-membered macrolide) tend to decrease the leakage of PLY via the impairment of LytA release and downregulation of *ply* gene transcription (19).

Therapeutic targeting of PLY is an attractive strategy for the treatment of pneumococcal diseases (20). In addition to ERY, several antibiotics, including clarithromycin (CLR; 14-membered macrolide) and clindamycin (CLI; lincosamide), may also decrease the production of PLY in S. pneumoniae (14, 21). However, the underlying mechanisms are poorly understood. In this study, we first compared the *in vitro* efficacy of these antibiotics and roxithromycin (ROX; 14-membered macrolide) on the release of PLY by MRSP. We also analyzed the mechanisms underlying the inhibition of PLY. Furthermore, the *in vivo* efficacy of the antibiotics was analyzed using a mouse model of intratracheal MRSP infection.

## RESULTS

### Treatment with CLR markedly decreases hemolytic activity via inhibition of PLY release in the supernatant of MRSP cell culture.

**FIG 1:**
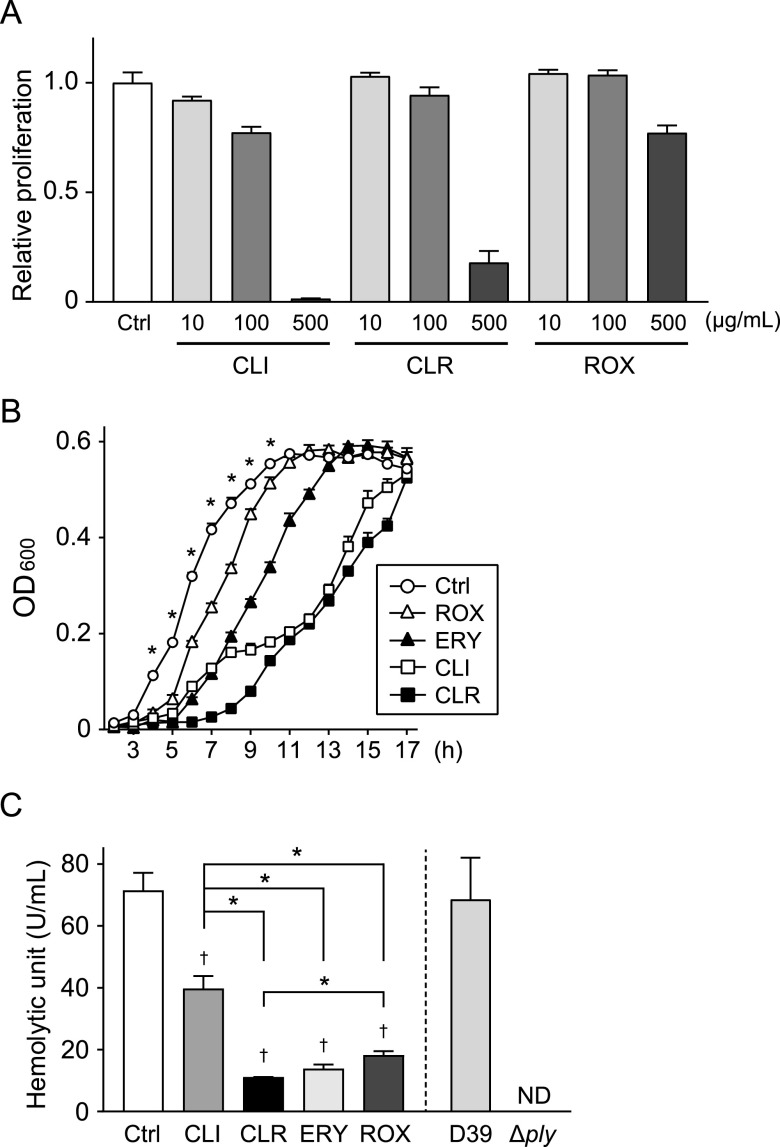
Effect of macrolides and lincosamide on the proliferation and hemolytic activity of macrolide-resistant S. pneumoniae (MRSP). (A) MRSP strain NU4471 was inoculated in TS broth and cultured with various concentrations of CLI, CLR, and ROX for 18 h at 37°C. The optical density (OD) of each well was measured at 600 nm. The data represent the mean ± SD of triplicate experiments. (B and C) MRSP strain NU4471 was cultured in the presence or absence of 5 μg/ml CLI, CLR, ERY, or ROX at 37°C. (B) The OD of each sample was measured at 600 nm at various time points. The data represent the mean ± SD of triplicate experiments and were evaluated using two-way analysis of variance with Tukey’s multiple-comparison test. *, Significantly different compared with all other antibiotic-treated groups at *P < *0.05. (C) The hemolytic activity of each cell-free supernatant was determined. Supernatants from S. pneumoniae wild-type strain D39 and the *ply* isogenic mutant (Δ*ply*) were used as controls. The data represent the mean ± SD of quadruplicate experiments and were evaluated using one-way analysis of variance with Tukey’s multiple-comparison test. †, Significantly different compared to the control at *P < *0.05. *, Significant difference between the indicated groups at *P < *0.05. CLI, clindamycin; CLR, clarithromycin; Ctrl, control; ERY, erythromycin; MRSP, macrolide-resistant Streptococcus pneumoniae; ND, not detected; OD, optical density; ROX, roxithromycin; SD, standard deviation; TS, tryptic soy.

Macrolide-resistant S. pneumoniae strain NU4471 is highly resistant to ERY (MIC, >1,000 μg/ml) (13). Figure 1A shows that the MICs of CLI, CLR, and ROX against this MRSP strain were >100 μg/ml, indicating the high resistance of this strain to these antibiotics. However, our previous study demonstrated that even sub-MICs of ERY retarded the growth of MRSP. Therefore, we investigated the effect of macrolides and CLI on MRSP bacterial growth. Figure 1B shows that all antibiotics used in this study significantly extended the lag phase of MRSP compared to that of the untreated control.

**FIG 2:**
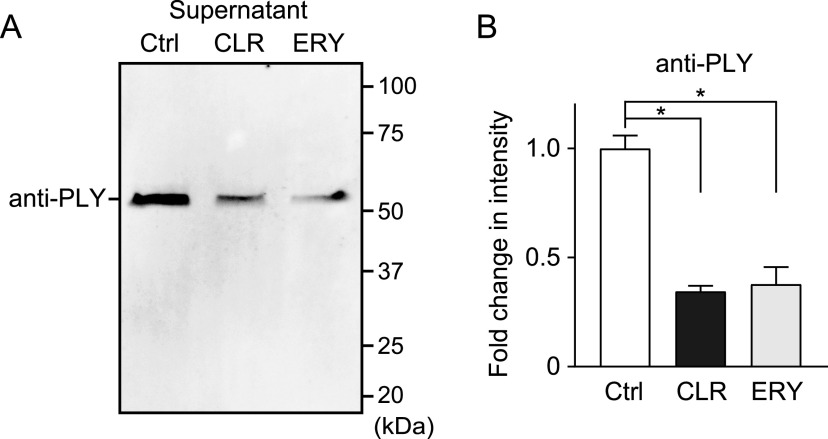
CLR and ERY treatment decreased PLY release in pneumococcal supernatant. (A) PLY protein levels in cell-free culture supernatants of 5 μg/ml CLR- or ERY-treated MRSP cultures were determined by Western blotting using an anti-PLY antibody. (B) The relative intensities of the bands were quantitatively analyzed. The data represent the mean ± SD of triplicate experiments and were evaluated using one-way analysis of variance with Tukey’s multiple-comparison test. *, Significantly different between the indicated groups at *P < *0.05. CLR, clarithromycin; Ctrl, control; ERY, erythromycin; PLY, pneumolysin; SD, standard deviation.

To investigate the inhibitory effect of these antibiotics on the release of PLY after the exclusion of their inhibitory effect on bacterial growth, MRSP were incubated in the presence or absence of antibiotics until they reached the stationary phase of growth (optical density at 600 nm [OD_600_], 0.55; the incubation periods of the untreated control, ROX, ERY, CLI, and CLR groups were 11, 12, 14, 17, and 17 h, respectively). Subsequently, the supernatants of the cultures were collected. Fig. S1 in the supplemental material shows that neither CLR nor ERY decreased pneumococcal CFU at the stationary growth phase. It has been reported that PLY is responsible for the hemolytic activity of pneumococci (22). Likewise, supernatant of S. pneumoniae wild-type strain D39 induced hemolysis, whereas hemolytic activity was completely abrogated in supernatant of *ply* isogenic mutant (Δ*ply*) (Fig. 1C). Therefore, we screened for antibiotics that inhibit the release of PLY using the hemolytic activity assay. All antibiotics significantly decreased the hemolytic activity of the MRSP supernatants (Fig. 1C). Among these, ERY markedly decreased its hemolytic activity, which is consistent with previous studies (19, 21). Additionally, CLR significantly decreased the hemolytic activity of the MRSP supernatant compared to both CLI and ROX and showed a similar or better inhibitory effect on the activity compared to ERY. CLR is a derivative of ERY and differs structurally from ERY in the substitution of an *O*-methyl group for the hydroxy group at position 6 of the lactone ring (23). In this study, we focused on the inhibitory effects of CLR on PLY production and its mechanisms in comparison to those of a control, ERY. Consistent with the findings in the hemolytic activity assay, Western blot analysis revealed that less PLY was detected in supernatants of cultures treated with CLR and ERY than in the untreated control (Fig. 2A and B). Figure S2A shows that the antibody was specific for PLY.

### CLR upregulates *lytA* gene transcription and increases pneumococcal autolysis.

**FIG 3:**
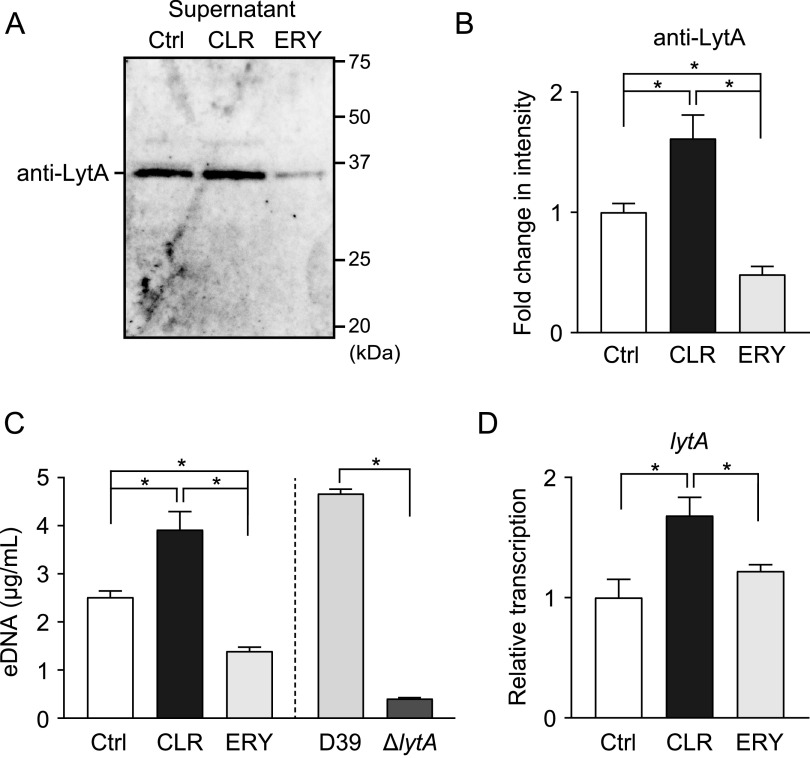
CLR increased *lytA* gene transcription and LytA release into culture supernatant. (A to C) MRSP NU4471 was incubated in the presence or absence of 5 μg/ml CLR or ERY until it reached the stationary phase of growth (OD_600_, 0.55). (A) LytA protein levels in cell-free culture supernatants were determined by Western blotting. (B) The relative intensities of the bands were quantitatively analyzed. (C) Pneumococcal eDNA in the culture supernatant was quantified using real-time PCR. Supernatant from S. pneumoniae wild-type strain D39 and the *lytA*-isogenic mutant (Δ*lytA*) were used as controls. (D) Real-time PCR was performed to quantify the transcription levels of *lytA* in MRSP treated with 5 μg/ml CLR or ERY for 2 h. The relative quantity of the gene was normalized to the relative quantity of 16S rRNA. In panels B to D, the data represent the mean ± SD of quadruplicate experiments and were evaluated using one-way analysis of variance with Tukey’s multiple-comparison test. *, Significant difference between the indicated groups at *P < *0.05. CLR, clarithromycin; Ctrl, control; eDNA, extracellular DNA; ERY, erythromycin; SD, standard deviation.

It has been reported that pneumococcal autolysin, LytA, contributes to pathogenesis by mediating autolysis and the release of intracellular toxins, such as PLY (24, 25). Our previous study demonstrated that ERY decreases PLY leakage by inhibiting LytA release (19). Therefore, we further examined whether CLR decreased the leakage of PLY via a similar mechanism. However, contrary to our expectation, LytA protein levels were significantly increased in the supernatant of CLR-treated MRSP culture compared to that of the untreated control (Fig. 3A and B). Figure S2B shows that the antibody was specific for LytA. Reportedly, pneumococcal autolysis causes DNA leakage into the bacterial supernatant (26, 27); thus, quantification of extracellular DNA (eDNA) using real-time PCR enabled the estimation of the autolytic activity of S. pneumoniae. Indeed, the concentration of eDNA in the supernatant of the *lytA* mutant (Δ*lytA*) was markedly decreased compared to that of wild-type strain D39 (Fig. 3C). Figure 3C also shows that the CLR treatment significantly increased the concentration of eDNA compared to that of the untreated control in the supernatant of MRSP strain NU4471. Furthermore, we found that CLR treatment upregulated *lytA* gene transcription (Fig. 3D). Collectively, the sub-MIC (5 μg/ml) of CLR inhibits PLY release despite the activation of pneumococcal autolysis.

### Compared to ERY, CLR significantly downregulates *ply* gene transcription and decreases hemolytic activity of pneumococcal cell lysate.

**FIG 4:**
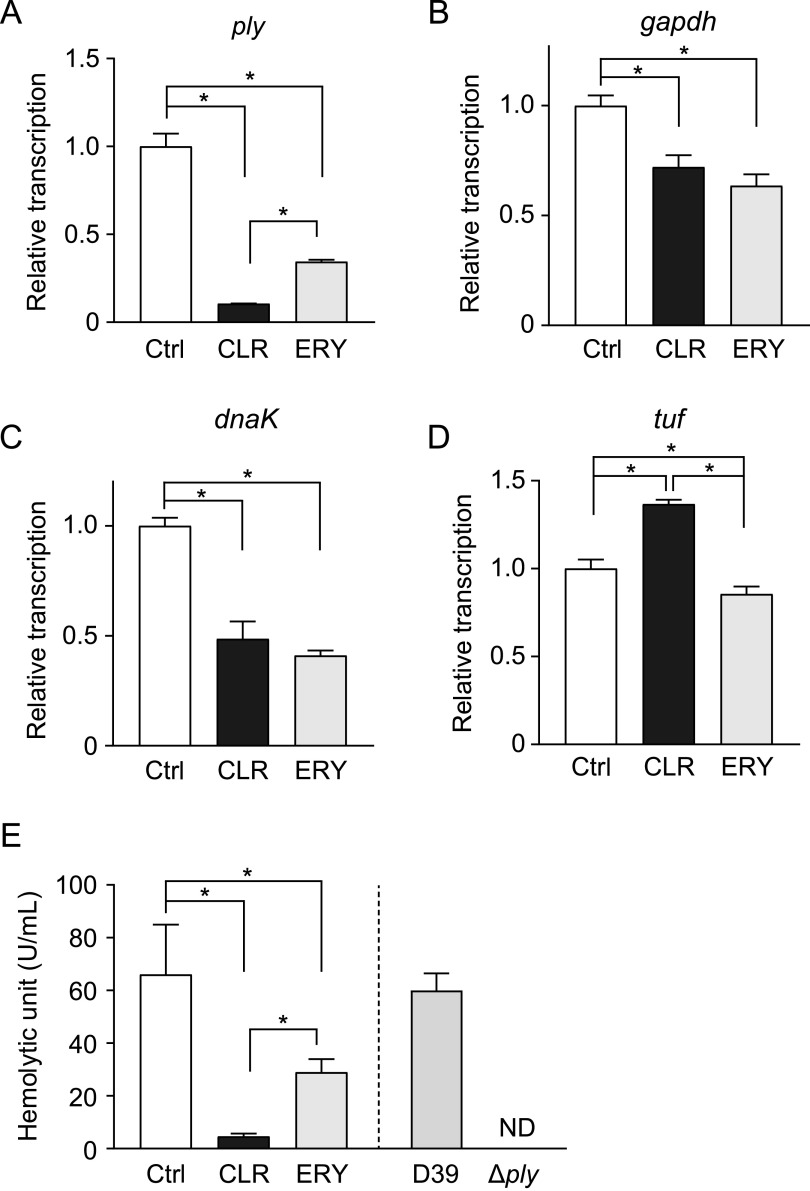
CLR decreased *ply* gene transcription and intracellular hemolytic activity of MRSP. (A to D) Real-time PCR was performed to quantify the transcription levels of (A) *ply*, (B) *gapdh*, (C) *dnaK*, and (D) *tuf* in MRSP treated with 5 μg/ml CLR or ERY for 2 h. The relative quantity of the gene was normalized to the relative quantity of 16S rRNA. (E) MRSP NU4471 was incubated in the presence or absence of 5 μg/ml CLR or ERY until it reached the stationary phase of growth (OD_600_, 0.55). Subsequently, the intracellular hemolytic activity of the culture was determined. S. pneumoniae wild-type strain D39 and the *ply* isogenic mutant (Δ*ply*) were used as controls. Data represent the mean ± SD of quadruplicate experiments and were evaluated using one-way analysis of variance with Tukey’s multiple-comparison test. *, Significant difference between the indicated groups at *P < *0.05. CLR, clarithromycin; Ctrl, control; ERY, erythromycin; MRSP, macrolide-resistant Streptococcus pneumoniae; ND, not detected; SD, standard deviation.

We hypothesized that the discrepancy between the activities of ERY and CLR might be due to the downregulation of *ply* gene transcription by CLR. Figure 4A shows that CLR significantly decreased the transcription of the gene compared to ERY. Next, we attempted to determine the PLY protein level in pneumococcal cells by Western blotting with anti-GAPDH antibody as the internal control; however, neither of the macrolides decreased the PLY protein level in the cells (data not shown), which was due to the significant downregulation of *gapdh* gene transcription by both CLR and ERY treatments (Fig. 4B). Additionally, macrolides significantly altered transcription of internal control gene candidates, *dnaK* and *tuf* (Fig. 4C and D). These results highlight the difficulty in identifying suitable internal control proteins in macrolide-treated pneumococci. Instead, we performed a hemolytic activity assay using bacterial cell lysates. Figure 4E shows that the hemolytic activity of CLR-treated pneumococcal lysates was significantly decreased compared to that of ERY-treated cell lysates. These findings indicate that the marked reduction in *ply* gene transcription results in the reduction of PLY release into the culture supernatant of CLR-treated MRSP.

### Exposure of other clinical MRSP isolates to CLR results in decreased hemolytic activity in the culture supernatant despite upregulation of eDNA leakage.

Next, we investigated the effect of macrolides on clinical MRSP isolates KM2412 and KM275, which were collected from the nasopharynx of patients with acute otitis media (3). MRSP strain KM2412 showed higher MIC values to macrolides (CLR and ERY MICs of 8 and 12 μg/ml, respectively) and harbored macrolide-resistant *ermB* and *mefA* genes, and strain KM275 (CLR and ERY MICs of 4 and 8 μg/ml, respectively) harbored the *ermB* gene only (Fig. S3A and B). Consistent with Fig. 1B, the sub-MIC (1 μg/ml) of CLR and ERY extended the lag phase of both MRSP isolates compared to those of the untreated control (Fig. S3C). However, this concentration of macrolides did not decrease the hemolytic activity of MRSP strain KM2412 or KM275 (Fig. S4A). Therefore, we next investigated the effect of higher concentrations (2 and 4 μg/ml) of macrolides on the hemolytic activity of KM2412. Figure S4B demonstrates that both 2 and 4 μg/ml of macrolides significantly decreased the hemolytic activity of the MRSP strain KM2412 supernatants. Additionally, 4 μg/ml CLR significantly decreased the hemolytic activity compared to 2 μg/ml CLR, indicating that a higher concentration of CLR is required to suppress the hemolytic activity of MRSP. Additionally, consistent with Fig. 3C, 4 μg/ml CLR significantly increased the concentration of eDNA compared to that in the untreated control in the supernatant of MRSP strain KM2412 (Fig. S4C). Taken together, these data show that the inhibitory effect of CLR on hemolytic activity is not limited to MRSP strain NU4471.

### Administration of CLR and ERY decrease PLY level in bronchoalveolar lavage fluid and ameliorate lung injury in an MRSP-infected mouse model.

**FIG 5:**
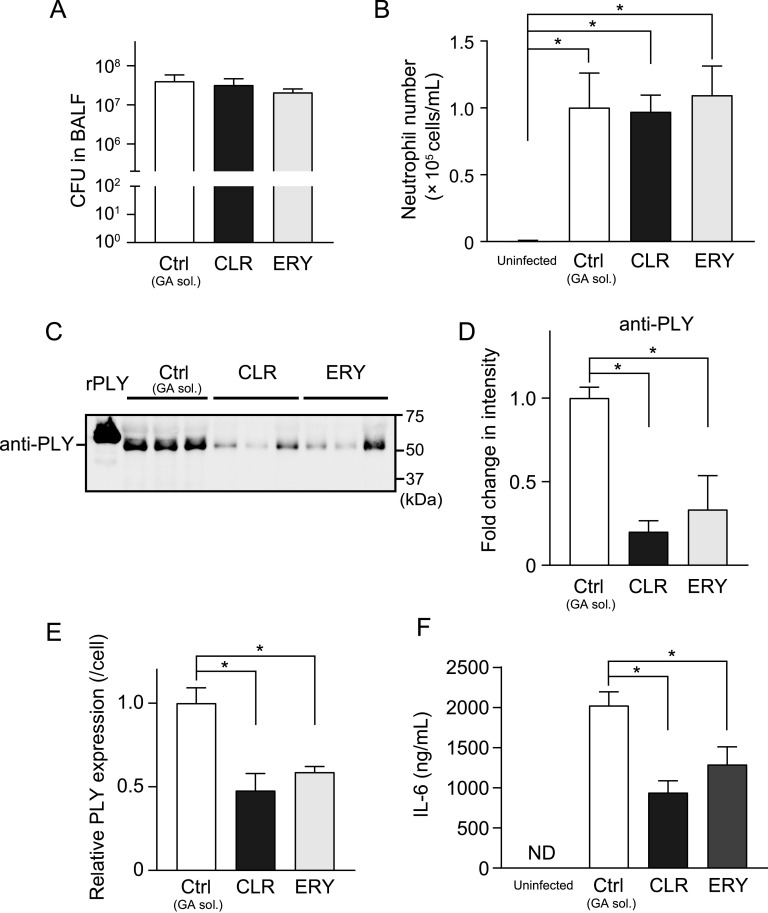
Administration of CLR or ERY decreased the PLY protein level without affecting pneumococcal CFU and neutrophil infiltration in BALF. BALB/c mice (eight mice in each group) were intratracheally infected with MRSP NU4471 (5 × 10^8^ CFU in 50 μl PBS). Uninfected mice were intratracheally administered 50 μl PBS only. CLR (150 mg/kg), ERY (150 mg/kg), or gum arabic solution (GA sol.; Ctrl) was administered orally to the infected mice every 12 h. The animals were sacrificed at 24 h postinfection. (A) BALF samples were plated onto blood agar plates and cultured aerobically to enumerate the number of recovered pneumococci. (B) The number of neutrophils was determined by flow cytometry based on the expression of both Ly6G and CD11b. (C) PLY protein levels in the BALF were determined by Western blotting. A representative Western blot image (three samples from each group) is shown. (D) Relative intensities of the bands were quantitatively analyzed. (E) Relative intensities of the bands were normalized to viable pneumococcal cell numbers in BALF. (F) IL-6 levels in BALF were determined using ELISA kits. In panels A, B, and D to F, the data represent the mean ± SEM and were evaluated using one-way analysis of variance with Tukey’s multiple-comparison test. *, Significant difference between the indicated groups at *P < *0.05. BALF, bronchoalveolar lavage fluid; CLR, clarithromycin; Ctrl, control; ELISA, enzyme-linked immunosorbent assay; ERY, erythromycin; GA sol., gum arabic solution; IL, interleukin; MRSP, macrolide-resistant Streptococcus pneumoniae; ND, not detected; rPLY, recombinant pneumolysin; SEM, standard error of the mean.

To compare the efficacy of CLR and ERY on MRSP-induced pneumonia *in vivo*, we orally administered either CLR or ERY (150 mg/kg every 12 h) to a mouse model of intratracheal MRSP infection. Figure 5A and B show that the administration of these macrolides did not directly result in a significant reduction in either viable MRSP counts or Ly-6G^+^ neutrophil numbers in the bronchoalveolar lavage fluid (BALF). However, PLY protein levels were significantly decreased in the BALF of both CLR- and ERY-treated mice compared to that of the untreated control mice (Fig. 5C to E), which is consistent with the *in vitro* data. PLY is recognized by Toll-like receptor 4 and induces proinflammatory cytokine production in macrophages (17). Consistent with this finding, we found that the reduction in PLY levels in CLR- and ERY-treated mice resulted in decreased interleukin-6 (IL-6) levels in the BALF of these mice compared to that of the untreated control group (Fig. 5F).

**FIG 6:**
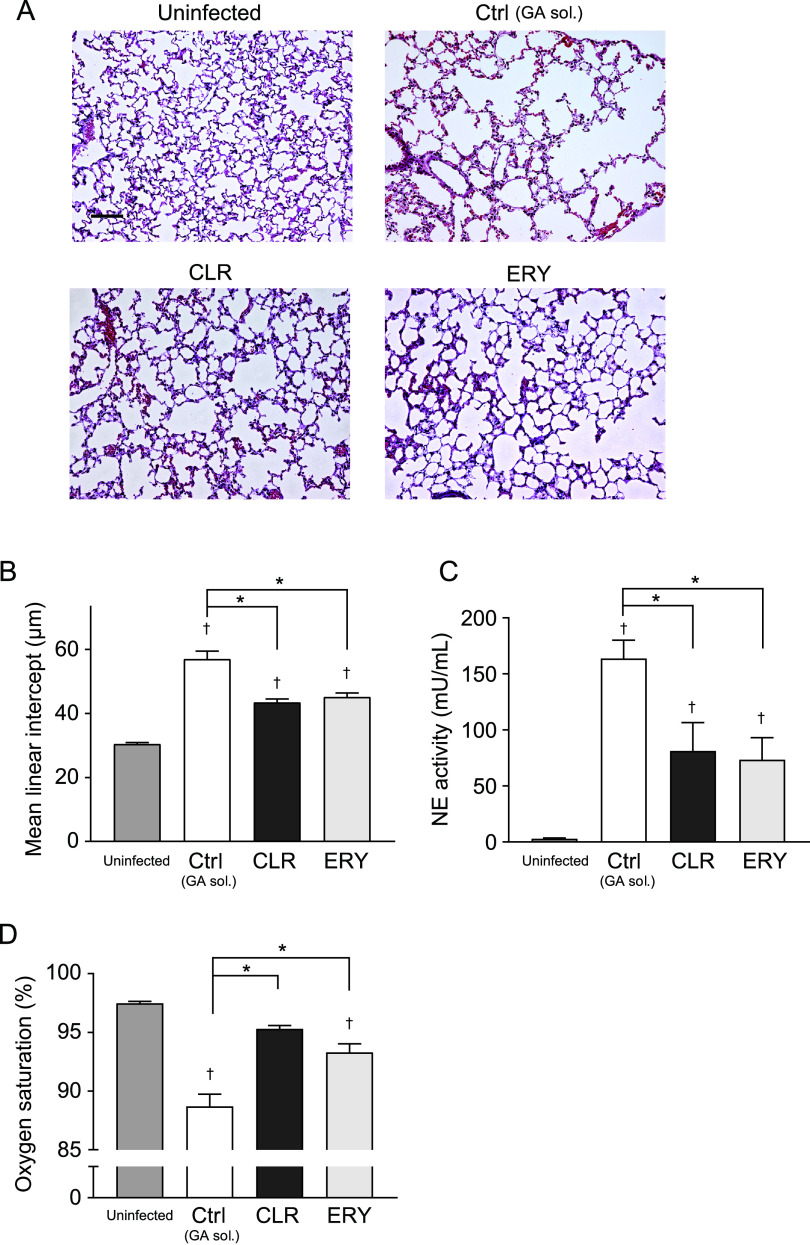
Administration of CLR or ERY decreased NE activity in BALF and ameliorated arterial oxygen saturation in a murine model of MRSP pneumonia. BALB/c mice (eight mice in each group) were intratracheally infected with MRSP NU4471 (5 × 10^8^ CFU in 50 μl PBS). Uninfected mice were intratracheally administered 50 μl PBS only. CLR (150 mg/kg), ERY (150 mg/kg), or gum arabic solution (GA sol.; Ctrl) was administered orally to the infected mice every 12 h. (A) Representative hematoxylin and eosin-stained lung tissue sections. Scale bar = 100 μm. (B) Mean linear intercept of alveolar septa. (C) NE activity in the BALF was determined by a method using an NE-specific substrate. (D) The arterial oxygen saturation was monitored. In panels B to D, the data represent the mean ± SEM and were evaluated using one-way analysis of variance with Tukey’s multiple-comparison test. †, Significantly different from the uninfected group at *P < *0.05. *, Significant difference between the indicated groups at *P < *0.05. BALF, bronchoalveolar lavage fluid; CLR, clarithromycin; Ctrl, control; ERY, erythromycin; GA sol., gum arabic solution; MRSP, macrolide-resistant *Streptococcus pneumoniae*; NE, neutrophil elastase; rPLY, recombinant pneumolysin; SEM, standard error of the mean.

**FIG 7:**
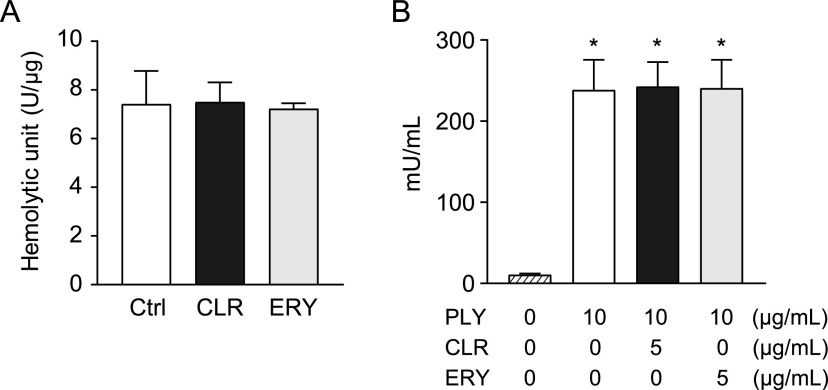
Neither CLR nor ERY affected the PLY-induced leakage of elastase from neutrophils. (A) Hemolytic activity of recombinant PLY was determined in the presence or absence of 5 μg/ml of CLR or ERY. (B) Human neutrophils (2 × 10^5^ cells in 200 μl) were exposed to recombinant PLY (10 μg/ml) in the presence or absence of 5 μg/ml CLR or ERY for 2 h. NE activity of the culture supernatant was evaluated using a human NE activity assay kit. Data represent the mean ± SD of triplicate experiments and were evaluated using one-way analysis of variance with Tukey’s multiple-comparison test. *, Significantly different from the untreated group at *P < *0.05. CLR, clarithromycin; Ctrl, control; ERY, erythromycin; NE, neutrophil elastase; PLY, pneumolysin; SD, standard deviation.

We evaluated lung tissue injury by performing a histological study. We found that the lungs of MRSP-infected mice exhibited enlarged air spaces with increased infiltration of inflammatory cells at 24 h postinfection (Fig. 6A). Additional morphometric analysis revealed that the intratracheal infection caused an 80% increase (approximately) in the mean linear intercept (*L*_m_; airspace size) of the infected mice compared to that of the uninfected group (Fig. 6B). In contrast, *L*_m_ was significantly decreased in CLR- and ERY-treated mice compared to that of the untreated control (Fig. 7A and B), indicating that both macrolides ameliorate lung injury in pneumococcal pneumonia. Reportedly, the destruction of lung parenchyma is due to the release of host proteolytic enzymes, such as neutrophil elastase (NE), from the neutrophils (28, 29). In this regard, we and other researchers have demonstrated that PLY induces neutrophil death and neutrophil extracellular trap formation, both of which lead to NE release (25, 30). Therefore, we measured NE activity in BALF using a specific substrate. Figure 6C shows that both CLR and ERY treatments decreased NE activity in BALF compared to that in the untreated control. Consistent with these findings, arterial oxygen saturation was significantly higher in both the CLR- and ERY-treated groups compared to that of the untreated control (Fig. 6D). There were no statistical differences in all parameters between the CLR- and ERY-treated groups *in vivo*.

### Macrolides do not decrease recombinant PLY-induced NE leakage from neutrophils *in vitro*.

We next investigated whether CLR and ERY directly inhibit the hemolytic activity of recombinant PLY. Figure 7A shows that neither CLR nor ERY altered the hemolytic activity. Since both CLR and ERY treatments decreased NE activity in BALF (Fig. 6C), we hypothesized that these macrolides may decrease NE activity in neutrophils. Human neutrophils were exposed to 10 μg/ml PLY in the presence or absence of CLR or ERY. However, treatment of these macrolides did not alter NE activity in the supernatant from PLY-treated neutrophils (Fig. 7B). These findings suggest that these macrolides do not inhibit pore-forming activity of PLY or NE activity in neutrophils.

## DISCUSSION

Previous studies have consistently indicated that several macrolides and lincosamides decrease PLY production or release by MRSP (13, 14, 21, 31). However, the current study indicates that macrolides significantly retarded pneumococcal growth, which was almost certainly related to decreased pneumolysin release within a certain time in the previous studies. We demonstrated that all macrolides used in this study and CLI were found to decrease the hemolytic activity of MRSP, regardless of its inhibitory activity against bacterial growth. Among these, CLR and ERY showed higher inhibitory effects on the hemolytic activity of MRSP, via the transcriptional downregulation of the *ply* gene. However, the effects of CLR on pneumococcal autolysin were different from those of ERY. ERY treatment impaired LytA release in MRSP and subsequently inhibited autolysis, which is consistent with our previous results (19), whereas CLR upregulated the transcription of the *lytA* gene and enhanced autolysis. We confirmed these findings in additional independent experiments (data not shown). Although less PLY is produced intracellularly due to reduced transcription of the *ply* gene with CLR treatment compared to ERY treatment, the increase in *lytA* gene transcription leading to enhanced autolysis with CLR treatment compared to ERY treatment results in the overall similar supernatant hemolytic activity and PLY concentrations. Overall similarity *in vitro* was consistent with no difference in effect observed between the macrolides demonstrated in the murine model of MRSP pneumonia. Specifically, the administration of both CLR and ERY significantly decreased the *L*_m_, and the PLY protein level and NE activity in BALF, and improved arterial oxygen saturation, without affecting the bacterial load in the murine model of MRSP pneumonia. These findings suggest that the macrolide-dependent inhibition of PLY reduces pneumococcal virulence.

PLY, a multifunctional pneumococcal virulence factor produced by all clinically relevant pneumococcal isolates (32), shows relatively limited variation between strains (33). PLY plays a role in the initial step of pneumococcal nasal colonization, which may be due to the PLY-induced disruption of the epithelial barrier (34). *In vitro* studies have demonstrated that PLY exhibits cytotoxicity against various cell types, including nasal and alveolar epithelial cells (15), and neutrophils are highly sensitive to PLY-induced cell lysis (25). Additionally, PLY induces NETosis and activates neutrophil extracellular trap formation (30). Following cell lysis or NETosis, neutrophils release NE, which subsequently induces the disruption of the alveolar epithelial barrier (29). Therefore, in addition to PLY, NE is considered a potential therapeutic target for various respiratory diseases, including pneumococcal pneumonia (35).

The serine protease, NE, plays a crucial role in the host defense against bacterial infections. As for S. pneumoniae, aminopeptidase N, which localizes in the pneumococcal cell wall, serves as a substrate for NE and contributes to the intracellular death of the organism in the neutrophils (36). However, the localized increase in NE activity in the lung has been correlated with acute lung injury via the degradation of cell-cell adhesion molecules (37, 38) and various extracellular matrix proteins (39). A previous study reported that the intratracheal administration of NE induced breakdown of alveoli followed by the elevation of *L*_m_ in a rat model (40). The results of this and previous studies indicate that intratracheal pneumococcal infection results in lung injury characterized by an increase in NE activity in BALF (41, 42). Although few studies have assessed the direct effects of PLY on lung injury, García-Suárez et al. reported that the ability of PLY to trigger inflammatory cell activity could play a major role in inducing *in vivo* lung injury, rather than inducing cytotoxic activity against lung tissue (43). These findings suggest that the PLY-induced leakage of NE from neutrophils is a major etiological factor in lung injury during pneumococcal pneumonia. In our study, since the macrolides did not directly decrease either neutrophil infiltration *in vivo* or recombinant PLY-induced NE leakage from neutrophils *in vitro*, we anticipate that the inhibitory effect of macrolides on PLY production mainly contributes to the reduction in the NE activity of BALF and subsequent amelioration of lung injury and arterial oxygen saturation in the murine model of intratracheal MRSP infection.

Macrolides inhibit bacterial growth by impeding the passage of newly synthesized polypeptides through the nascent peptide exit tunnel of the bacterial ribosome. Previously, macrolides were considered to indiscriminately block the elongation of every protein during the early stages of translation. In contrast, a recent study has shown that macrolides differentially inhibit the synthesis of individual proteins. Kannan et al. reported that the most prevalent motif at the ERY-induced translation arrest sites conformed to the consensus [R/K]X[R/K] (44). Therefore, it is possible that, in addition to the downregulation of *ply* gene transcription, macrolides may also inhibit PLY protein synthesis at an intermediate stage at the KD_187_K motif. In contrast, in our study, we found that the effects of CLR and ERY on the transcription of the *lytA* gene were different; CLR upregulated *lytA* gene transcription in MRSP, whereas ERY showed no effect. The precise regulation of bacterial gene transcription remains poorly understood. Further *in vitro* studies are needed to evaluate the transcriptional regulation of virulent bacterial genes by macrolides.

Although the main goal of treatment for CAP patients is the elimination of the causative organism, our findings indicate that macrolide monotherapy does not significantly decrease the CFU of MRSP in BALF. To cover a wider spectrum and elicit a synergistic effect, combination antibiotic therapy with different mechanisms of action have sometimes been used for the treatment of pneumonia. In this regard, several studies have demonstrated the efficacy of macrolide and β-lactam combination therapy in mouse models of severe pneumonia caused by S. pneumoniae, irrespective of their antimicrobial susceptibility patterns (45, 46). Clinical studies have also indicated that combination therapy leads to considerably reduced mortality among CAP patients (47). Although exposure to β-lactams can cause the lysis of S. pneumoniae cells with increased release of PLY (48), macrolides are considered to compensate for the shortcomings of β-lactams. Additionally, the immunomodulatory effects induced by macrolides may contribute to their clinical efficacy in the treatment of MRSP pneumonia, because macrolides are also effective against noninfectious lung inflammation (49). Therefore, it is difficult to clearly separate the effects of macrolides on host immunity and pneumococcal virulence factors. These findings represent a limitation in the use of animal infection models for evaluating the efficacy of macrolides. Further studies are required to clarify the mechanisms of macrolide action in pneumococcal diseases.

In conclusion, through this study, we have demonstrated that both CLR and ERY reduce the production of PLY in MRSP *in vitro*. Although the effects of these macrolides on pneumococcal autolysis are different, both macrolides decrease the PLY protein level in the BALF of a mouse model with MRSP pneumonia and ameliorate lung injury. These data reinforce the benefits of macrolides on the clinical outcomes of pneumococcal pneumonia.

## MATERIALS AND METHODS

### Bacteria, mice, and reagents.

A clinical isolate of macrolide-resistant S. pneumoniae NU4471 (serotype 19; MIC of penicillin G and ERY were 2 and >1,000 μg/ml, respectively), which harbors macrolide-resistant *ermB* and *mefA* genes, was mainly used in this study (13). Additionally, clinical MRSP isolates KM2412 and KM275, which were collected from the nasopharynx of patients with acute otitis media, were used (refer to supplemental material). All pneumococcal strains were grown in tryptic soy (TS) broth (Becton Dickinson, Franklin Lakes, NJ, USA) under aerobic conditions at 37°C. Male BALB/c mice (10- to 12-week-old mice) were obtained from Nihon CLEA (Tokyo, Japan). The mice were maintained under standard conditions in accordance with the institutional guidelines. All animal experiments were approved by the Institutional Animal Care and Use Committee of Niigata University (SA00451). CLI, CLR, ERY, and ROX were purchased from Tokyo Chemical Industry (Tokyo, Japan). All antibiotics were dissolved in ethanol and diluted with sterile water for subsequent *in vitro* experiments. Rabbit anti-pneumococcal LytA antibody was produced by Eurofins Genomics K.K. (Tokyo, Japan), as described previously (19). The expression and purification of His-tagged recombinant PLY was performed as described previously (25).

### Effect of macrolides on the growth of MRSP.

First, 100-μl aliquots of S. pneumoniae NU4471, grown until the exponential phase, were inoculated in 10 ml of TS broth. To determine the sensitivity of MRSP to macrolides and lincosamide, various concentrations (10 to 500 μg/ml) of CLI, CLR, and ROX were added to the bacterial cultures and incubated at 37°C for 18 h. Bacterial growth was determined by measuring the optical density (OD) of the cultures at a wavelength of 600 nm using a miniphoto 518R (Taitec, Tokyo, Japan). To determine the inhibitory effect of sub-MICs of macrolides and lincosamide on the growth of MRSP, 0.05% ethanol (control) or 5 μg/ml of ROX, ERY, CLI, and CLR were separately added to the bacterial cultures, and the cultures were incubated at 37°C. Bacterial growth was monitored by continuously measuring the OD of the bacterial culture at a wavelength of 600 nm.

### Hemolytic assay.

S. pneumoniae NU4471 cultures were grown in TS broth in the presence of 0.05% ethanol or 5 μg/ml of ROX, ERY, CLI, and CLR until they reached the early stationary phase (OD_600_, 0.55). Subsequently, the bacterial culture supernatants were collected by centrifugation at 5,000 × *g* for 10 min and filtration using a 0.22-μm pore size syringe filter (GVS Filter Technology, Indianapolis, IN, USA). These bacterial supernatants were used for Western blot and real-time PCR analyses, as described below. Bacterial cell pellets were resuspended in 1 ml phosphate-buffered saline (PBS) and homogenized with a MagNA Lyser instrument (Roche Diagnostics, Indianapolis, IN, USA) using 0.1-mm silica beads (MP Biomedicals, Solon, OH, USA). Subsequently, they were centrifuged at 5,000 × *g* for 10 min, and the resulting supernatant was used as the pneumococcal cell lysate. The culture supernatant and cell lysates were mixed with a 1% suspension of sheep erythrocytes (Nippon Bio-Test Laboratories, Tokyo, Japan) in PBS and incubated at 37°C for 30 min. Subsequently, they were centrifuged at 2,000 × *g* for 10 min. The absorbance of the supernatant was measured at 571 nm using a microplate reader (Thermo Fisher Scientific, Waltham, MA, USA). A hemolytic unit is defined as the dilution that results in 50% lysis of the erythrocyte suspension (50).

### Western blot analysis.

Pneumococcal culture supernatants were collected as described above, mixed with SDS sample buffer, separated by SDS-PAGE using 12% polyacrylamide gel (Bio-Rad Laboratories, Hercules, CA, USA), and transferred to polyvinylidene difluoride membranes (Merck Millipore, Burlington, MA, USA). The membranes were probed with anti-PLY antibody (Abcam, Cambridge, UK) or anti-LytA antibody and incubated with a horseradish peroxidase (HRP)-conjugated secondary antibody (Cell Signaling Technology, Danvers, MA, USA). Subsequently, the membranes were incubated with ECL Select reagent (GE Healthcare, Little Chalfont, UK) and analyzed using a chemiluminescence detector (Fujifilm, Tokyo, Japan). The intensity of the signal was quantified using Image Studio software version 5.2 (LI-COR Bioscience, Lincoln, NE, USA).

### Quantification of pneumococcal gene transcription and eDNA by real-time PCR.

MRSP strain NU4471 was cultured in TS broth until it reached the exponential growth phase (OD_600_, 0.2). Subsequently, the culture was incubated in the presence of 0.05% ethanol or 5 μg/ml CLR or ERY for 2 h at 37°C. Bacterial pellets were resuspended in TRI reagent (Molecular Research Center, Cincinnati, OH, USA) and homogenized with a MagNA Lyser instrument (Roche Diagnostics) using 0.1-mm silica beads. Bacterial RNA was then isolated using a Direct-zol RNA kit (Zymo Research, Irvine, CA, USA). RNA was reverse transcribed using SuperScript IV VILO master mix (Thermo Fisher Scientific) according to the manufacturer’s instructions. Subsequently, quantitative real-time PCR was performed on a StepOnePlus real-time PCR system (Thermo Fisher Scientific) using the cDNA as the template and the Fast SYBR green master mix (Thermo Fisher Scientific) according to the manufacturer’s instructions. The primer sequences for *lytA*, *ply*, *gapdh*, and 16S rRNA (control) were obtained from previous studies (19, 51). The primer sequences for *dnaK* and *tuf* are shown in Table 1.

**TABLE 1:** Primer sequences for real-time PCR

Target	Sequence (5′–3′)	
*dnaK*	Forward	TGGTGGTGACGACTTTGACC
*dnaK*	Reverse	CGCTTTTTCAGCCGCATCTT
*tuf*	Forward	CCCAGAACCAGAACGTGACA
*tuf*	Reverse	TACCACGGTCGATACGTCCT

To determine the concentration of eDNA in the collected pneumococcal culture supernatants, we performed absolute quantification using real-time PCR. To prepare a standard curve, pneumococcal DNA was extracted and purified using the GenElute bacterial genomic DNA kit (Sigma-Aldrich) according to the manufacturer’s instructions. The DNA concentration was determined using a NanoDrop Lite spectrophotometer (Thermo Fisher Scientific). Subsequently, quantitative real-time PCR was performed using the Fast SYBR green master mix. The primers used in the PCR were designed to target a fragment of the PLY-encoding gene, as described previously (26).

### Human NE activity assay.

Heparinized blood was obtained from three healthy donors, and human neutrophils were isolated as previously described (52). Briefly, whole blood was layered onto Polymorphprep (Axis Shield, Dundee, UK) in a 1:1 ratio and centrifuged at 500 × *g* for 30 min. The layers containing neutrophils were collected, and residual red blood cells were lysed using ACK lysing buffer (Lonza, Basel, Switzerland). Subsequently, human neutrophils (2 × 10^5^ cells in 200 μl) were exposed to recombinant PLY (10 μg/ml) in the presence or absence of 5 μg/ml CLR or ERY for 2 h. The culture supernatant was evaluated for NE activity using a human NE activity assay kit (Cayman Chemical, Ann Arbor, MI, USA) according to the manufacturer’s instructions. The experimental protocol adhered to the principles of the Declaration of Helsinki and was approved by the Institutional Review Board of Niigata University. Experiments were carried out in accordance with approved guidelines. Informed consent was obtained from all donors prior to their inclusion in the study (permit no. 2018-0075).

### Intratracheal infection of MRSP *in vivo*.

Following the administration of anesthesia (a mixture of medetomidine hydrochloride [Orion Corporation, Espoo, Finland], midazolam [Sandoz, Tokyo, Japan], and butorphanol [Meiji Seika Pharma Co., Ltd.]), BALB/c mice were intratracheally infected with S. pneumoniae strain NU4471 (5.0 × 10^8^ CFU in 50 μl PBS) using a MicroSprayer aerosolizer (Penn-Century, Inc., Philadelphia, PA, USA) as described previously (53). CLR or ERY powder was dissolved in 5% gum arabic solution. Pharmacokinetic analysis of CLR in mice has demonstrated that the regimen of 150 mg/kg twice a day is representative of human pharmacokinetics (54). Therefore, CLR (150 mg/kg), ERY (150 mg/kg), or 5% gum arabic solution (control) was administered via oral gavage to the infected mice at 0 and 12 h postinfection. At 24 h postinfection, each mouse was briefly anesthetized with isoflurane using an inhalational anesthetizer system (Natsume Seisakusho, Tokyo, Japan) (55). Subsequently, the arterial oxygen saturation was measured using MouseOx Plus (STARR Life Sciences Corp., Oakmont, PA, USA) according to manufacturer’s instructions, and the mice were sacrificed immediately. To obtain BALF, 1 ml PBS was introduced into mouse lungs and slowly aspirated (56). BALF samples were plated onto 5% sheep blood agar plates and cultured aerobically to enumerate the recovered CFU. Subsequently, BALF samples were centrifuged at 500 × *g*, and the cell-free supernatant was used for subsequent NE activity assay, cytokine analysis, and Western blot analysis.

Mouse NE activity in BALF was determined by a method using the NE-specific substrate, N-methoxysuccinyl-Ala-Ala-Pro-Val p-nitroanilide (Merck Millipore), as described previously (53). IL-6 levels in BALF were determined using enzyme-linked immunosorbent assay (ELISA) kits (BioLegend, San Diego, CA, USA). Additionally, PLY protein levels in BALF samples were determined by Western blot analysis using anti-PLY antibody (Abcam).

The cell pellet of BALF was analyzed for neutrophil count. Briefly, cells were treated with anti-CD16/32 antibody (Thermo Fisher Scientific) to block nonspecific binding of immunoglobulins to Fc receptors, stained with anti-CD11b-allophycocyanin and Ly-6G-phycoerythrin antibodies (Thermo Fisher Scientific), and fixed with 4% paraformaldehyde (Fujifilm Wako Pure Chemical Corporation, Osaka, Japan). Neutrophils were identified by the expression of both Ly-6G and CD11b on a NovoCyte flow cytometer using the NovoExpress software (ACEA Biosciences, San Diego, CA, USA).

For histological examination, the lung tissue samples were fixed with 4% paraformaldehyde. Paraffin-embedded sections were prepared by Biopathology Institute Co., Ltd. (Oita, Japan). These sections were stained with hematoxylin and eosin (Sakura Finetek Japan Co., Ltd., Tokyo, Japan) and observed under a BioRevo BZ-9000 microscope (Keyence, Osaka, Japan).

### Calculation of *L*_m_.

To calculate *L*_m_, 5 nonoverlapping regions/mouse were systematically acquired from each section. Briefly, six equally distributed horizontal and six vertical lines (lattice square grid) were laid over the digitized image of a hematoxylin and eosin-stained section. For each line, the intercepts with the tissue structures were counted. *L*_m_ is calculated as the ratio between the product of the number of times the traverses are placed on the lung and the length of the traverses, and the sum of all the intercepts (57). Only regions without bronchioles, large blood vessels (>50 μm), and other nonairway sections were selected.

### Statistical analysis.

Statistical analysis of the data was performed using one- or two-way analysis of variance with Tukey’s multiple-comparison test, using Prism Software version 7.05 (GraphPad Software, Inc., La Jolla, CA, USA).
