# Supplementary material for: Clarithromycin Inhibits Pneumolysin Production via Downregulation of ply Gene Transcription despite Autolysis Activation
Source: Microbiol Spectr. 2021 Sep 1;9(2):e00318-21. doi: 10.1128/Spectrum.00318-21 (PMC8557819; doi:10.1128/Spectrum.00318-21)
Supplement: SUPPLEMENTAL FILE 1 — Supplemental material. Download SPECTRUM00318-21_Supp_1_seq9.pdf, PDF file, 0.7 MB. [file spectrum00318-21_supp_1_seq9.pdf]

Clarithromycin inhibits pneumolysin production via downregulation of *ply* gene transcription despite autolysis activation

Hisanori Domon[a,b], Toshihito Isono[a], Takumi Hiyoshi[a,b,c], Hikaru Tamura[a,b,c], Karin Sasagawa[a,c], Tomoki Maekawa[a,b,c], Satoru Hirayama[a], Katsunori Yanagihara[d], Yutaka Terao[a,b]#

[a]Division of Microbiology and Infectious Diseases, Niigata University Graduate School of Medical and Dental Sciences, Niigata, Japan.

[b]Center for Advanced Oral Science, Niigata University Graduate School of Medical and Dental Sciences, Niigata, Japan.

[c]Division of Periodontology, Niigata University Graduate School of Medical and Dental Sciences, Niigata, Japan.

[d]Department of Laboratory Medicine, Nagasaki University Graduate School of Biomedical Sciences, Nagasaki, Japan.

#Address for correspondence to Yutaka Terao, terao@dent.niigata-u.ac.jp

Supporting Methods

Construction of *ply* and *lytA* mutant strains of *S. pneumoniae*

Inactivation of the *ply* and *lytA* genes in *S. pneumoniae* D39 was performed by double cross-over recombination, referring to the method of Ogawa *et al* (1). Briefly, approximately one kilobase upstream and downstream of the open reading frame (ORF) regions of *ply* and *lytA* were amplified by PCR from the genomic DNA of the strain D39. Similarly, the ORF region of spectinomycin resistance gene was amplified from plasmid pSET4s (2). The amplified products were used to construct antibiotic resistance cassettes with long flanking regions homologous to the target genes by overlap extension PCR (3). The PCR products were used for the transformation of D39 competent cells. For the preparation of the competent cells, the bacteria were grown in Todd-Hewitt broth with 0.5% yeast extract (THY) to OD_{600} = 0.25, and cultures were frozen in 15% glycerol at −80°C. For transformation, 50 µL of frozen competent cells were thawed and diluted with 1 mL of pre-warmed competent medium (Tryptic soy broth [pH 8.0], 10% glycerol, 0.16% bovine serum albumin, 0.01% CaCl2) containing 200 ng of competence stimulating peptide-1 (EMRLSKFFRDFILQRKK; synthesized by Eurofins Genomics K.K., Tokyo, Japan) and 1 µg of purified PCR product. Transformation reaction was performed for 3 h at 37°C, and small aliquots were plated on THY agar plates supplemented with 100 µg/mL spectinomycin and incubated for 24 h at 37°C. Inactivation of the *ply* and *lytA* genes was confirmed by PCR using primers homologous to the ORF region of the spectinomycin resistance gene and the primers upstream and downstream of the target genes. All primers used for PCR are shown in Supplemental Table S1.

References

1. Ogawa M, Matsuda R, Takada N, Tomokiyo M, Yamamoto S, Shizukuishi S, Yamaji T, Yoshikawa Y, Yoshida M, Tanida I, Koike M, Murai M, Morita H, Takeyama H, Ryo A, Guan JL, Yamamoto M, Inoue JI, Yanagawa T, Fukuda M, Kawabe H, Ohnishi M. 2018. Molecular mechanisms of *Streptococcus pneumoniae*-targeted autophagy via pneumolysin, Golgi-resident Rab41, and Nedd4-1-mediated K63-linked ubiquitination. Cell Microbiol 20:e12846.
2. Takamatsu D, Osaki M, Sekizaki T. 2001. Thermosensitive suicide vectors for gene replacement in *Streptococcus suis*. Plasmid 46:140-8.
3. Yamamoto S, Izumiya H, Morita M, Arakawa E, Watanabe H. 2009. Application of λ Red recombination system to *Vibrio cholerae* genetics: simple methods for inactivation and modification of chromosomal genes. Gene 438:57-64.

Figure S1

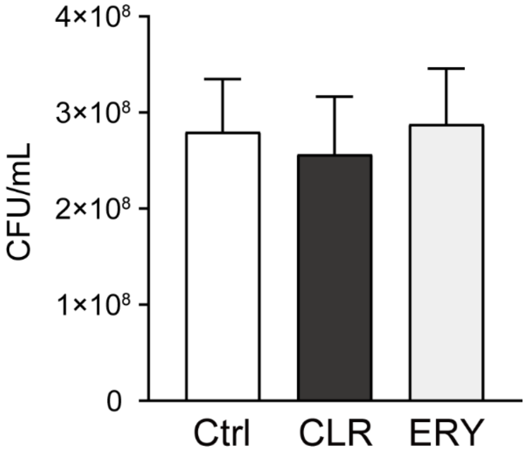

Supplemental Figure S1. CFU values of macrolide-treated MRSP strain NU4471

MRSP strain NU4471 was incubated in the presence or absence of 5 μg/mL CLR or ERY until it reached the stationary phase of growth (OD_{600} = 0.55). Samples were plated onto blood-agar plates and cultured aerobically for enumerating recovered CFU. Data represent the mean ± SD of triplicate experiments and were evaluated using one-way analysis of variance with Tukey's multiple-comparisons test.

CLR, clarithromycin; CFU, colony forming units; Ctrl, control; ERY, erythromycin; OD, optical density; MRSP, macrolide-resistant *Streptococcus pneumoniae*; SD, standard deviation

Figure S2

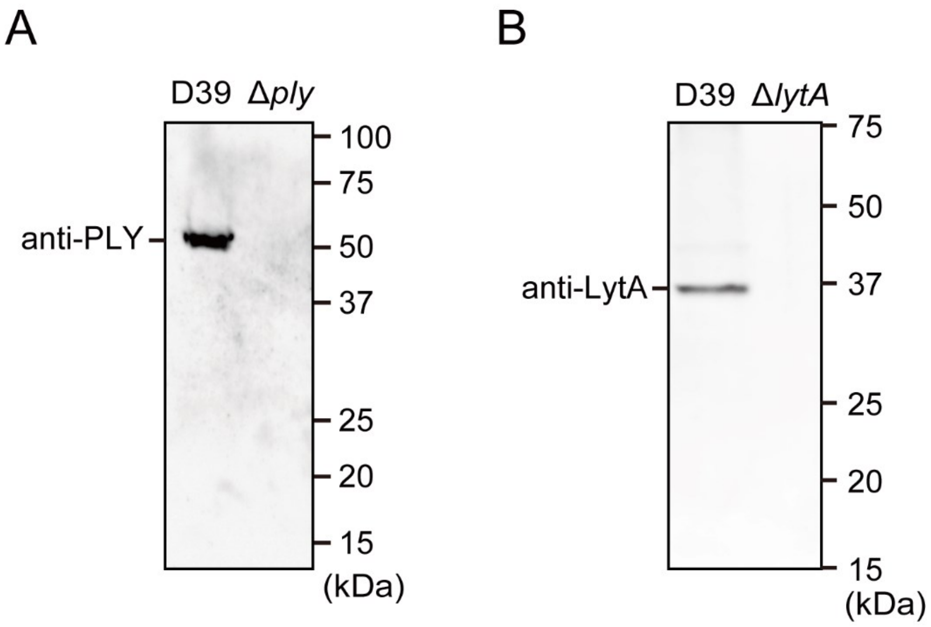

Supplemental Figure S2. Western blotting images showing the expression of PLY and LytA in the pneumococcal supernatant.

(A) Expression of PLY protein in the supernatant from *S. pneumoniae* wild-type strain D39 and *ply*-isogenic mutant (Δ*ply*) was determined by Western blotting. (B) Expression of LytA protein in the supernatant from *S. pneumoniae* wild-type strain D39 and *lytA*-mutant (Δ*lytA*) was determined by Western blotting.

Figure S3

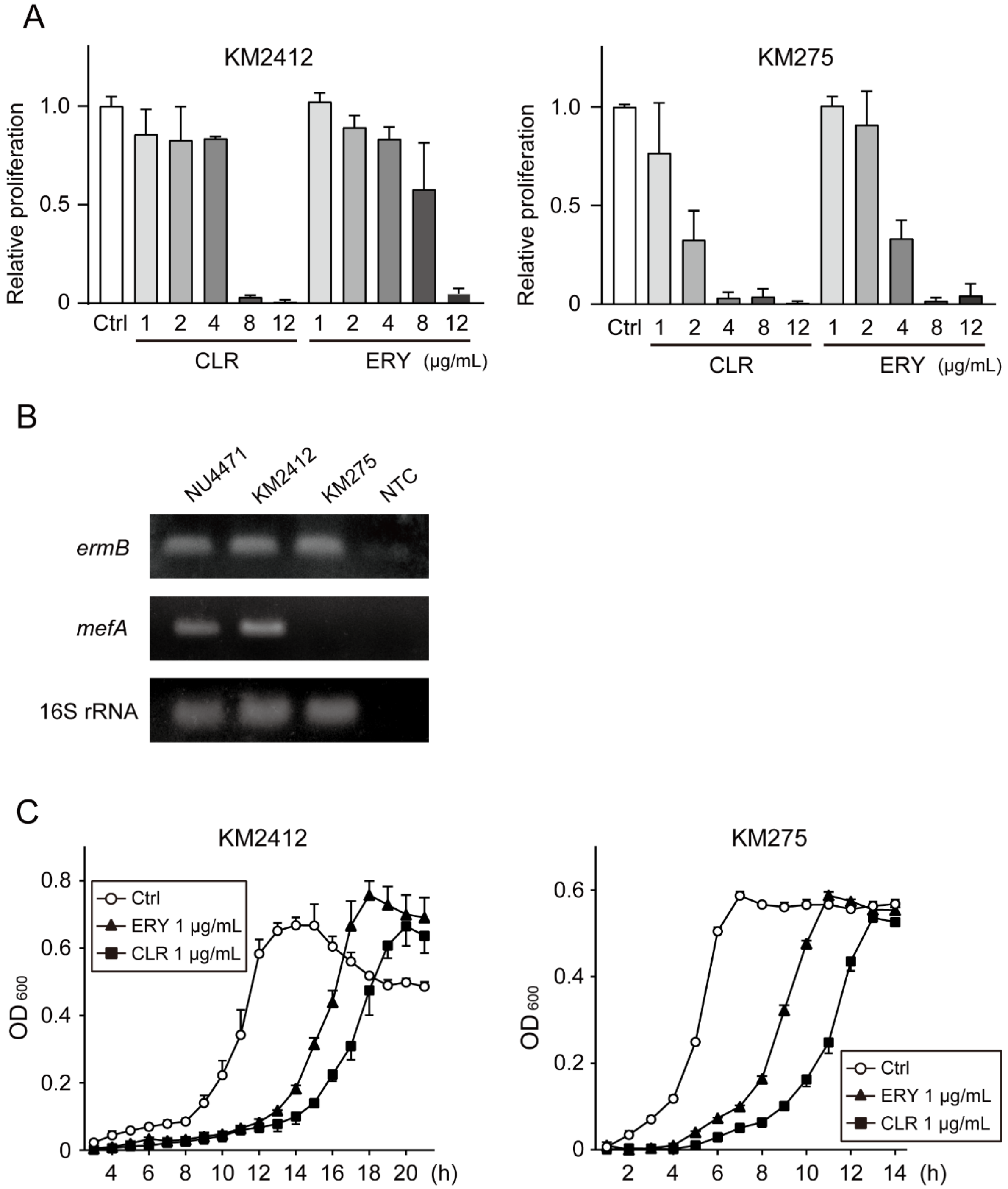
A
KM2412
Relative proliferation
1.0
0.5
0
Ctrl 1 2 4 8 12 1 2 4 8 12
CLR
ERY (μg/mL)
KM275
Relative proliferation
1.0
0.5
0
Ctrl 1 2 4 8 12 1 2 4 8 12
CLR
ERY (μg/mL)
B
NU4471
KM2412
KM275
NTC
ermB
mefA
16S rRNA
C
KM2412
OD 600
0.8
0.6
0.4
0.2
0
Ctrl
ERY 1 μg/mL
CLR 1 μg/mL
4 6 8 10 12 14 16 18 20 (h)
KM275
OD 600
0.6
0.4
0.2
0
Ctrl
ERY 1 μg/mL
CLR 1 μg/mL
2 4 6 8 10 12 14 (h)

Supplemental Figure S3. Characteristics of macrolide resistant *S. pneumoniae* isolates KM2412 and KM275.

(A) MRSP strain KM2412 and KM275 was inoculated in tryptic soy broth and cultured with various concentrations of CLR and ERY for 24 h at 37°C. The optical density (OD) of each well was measured at 600 nm. Data represent the mean ± SD of triplicate experiments. (B) MRSP strains NU4471 (positive control), KM2412, and KM275 was cultured in tryptic soy broth until they reached the stationary phase of growth. The bacterial cells were resuspended in TRI Reagent (Molecular Research Center, Cincinnati, OH, USA) and homogenized with a MagNA Lyser instrument using 0.1 mm silica beads followed by RNA isolation using a Direct-zol RNA kit (Zymo Research, Irvine, CA, USA). The RNA was reverse transcribed using SuperScript VILO Master Mix (Thermo Fisher Scientific), and RT-PCR was performed to analyze the transcription of macrolide-resistant *ermB* and *mefA* gene using a 2720 Thermal Cycler (Thermo Fisher Scientific, Waltham, MA, USA). The primer sequences are listed below (Supplemental Table S2). The PCR conditions were 94°C for 20 s, 60°C for 20 s, and 72°C for 15 s, with a total of 35 cycles. (C) CLR and ERY (1 μg/mL) were separately added to the MRSP strains KM2412 and KM275 cultures and the cultures were incubated at 37 °C. Bacterial growth was monitored by continuously measuring the OD of the bacterial culture at a wavelength of 600 nm. Data represent the mean ± SD of triplicate experiments.

CLR, clarithromycin; Ctrl, control; ERY, erythromycin; MRSP, macrolide-resistant *Streptococcus pneumoniae*; SD, standard deviation

Figure S4

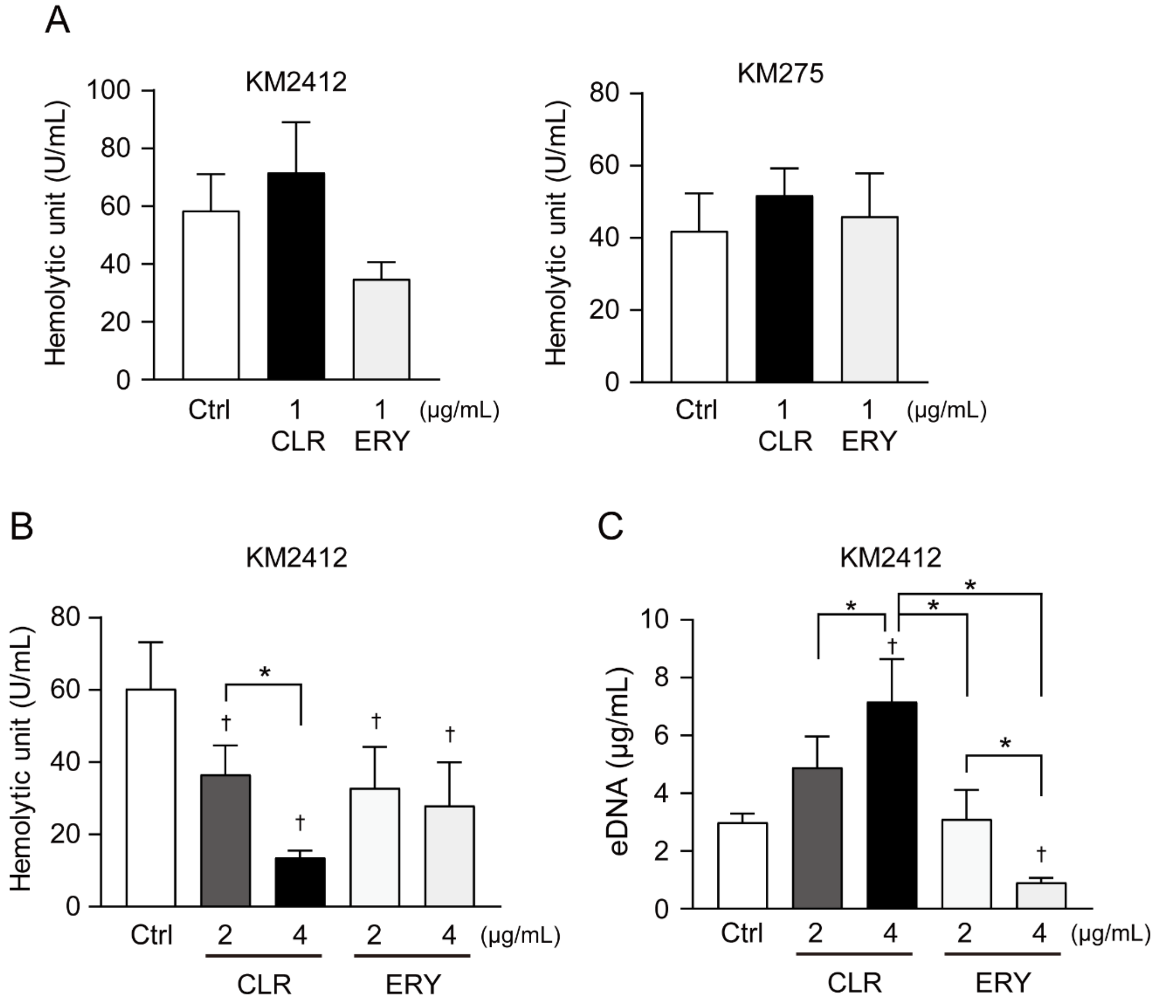

Supplemental Figure S4. Higher concentrations of macrolides decreased hemolytic activity in the supernatants of MRSP strain KM2412.

(A) MRSP strains KM2412 and KM275 were cultured in the presence or absence of 1 μg/mL CLR or ERY until they reached the stationary phase of growth (KM2412; OD_{600} = 0.7–0.75, KM275; OD_{600} = 0.55). The hemolytic activity of each cell-free supernatant was determined. (B) MRSP strain KM2412 was cultured in the presence or absence of 2–4 μg/mL CLR or ERY until it reached the stationary phase of growth. The hemolytic activity of each cell-free

supernatant was determined. (C) Pneumococcal eDNA in the culture supernatant of macrolide-treated MRSP strain KM2412 was quantified using real-time PCR. Data represent the mean ± SD of quadruplicate experiments and were evaluated using a one-way analysis of variance with Tukey's multiple-comparisons test. †Significantly different compared to the control at $P < 0.05$. *Significant difference between the indicated groups at $P < 0.05$.

CLR, clarithromycin; Ctrl, control; eDNA, extracellular DNA; ERY, erythromycin; OD, optical density; MRSP, macrolide-resistant *Streptococcus pneumoniae*; SD, standard deviation

Supplemental Table S1. Primer sequences for gene inactivation of *S. pneumoniae* D39.

Primers	Sequence (5' to 3')
Primers for amplification of upstream and downstream of the *ply* gene	
ply_up1000_F	ATGCGAAGGTCTTATCTCAGAG
anti_Spc+ply_up1000_R	CGTATGTATTCAAATATATCCTCCTCACCTTCTACCTCCTAATAAGTTCCTGG
anti_Spc+ply_dn1000_F	CTATAAACTATTTAAATAACAGATTAAAAAAATTATAAGAGAGGAGAATGCTTGCG
ply_dn1000_R	AACCTTCGTAACGTCCTTCTG
Primers for amplification of upstream and downstream of the *lytA* gene	
lytA_up1000_F	CTTGGTTAATGTGGTTCTGGATCTC
anti_Spc+lytA_up1000_R	CGTATGTATTCAAATATATCCTCCTCACATTCTACTCCTTATCAATTAAAACAACTCATTTTTTAC
anti_Spc+lytA_dn1000_F	CTATAAACTATTTAAATAACAGATTAAAAAAATTATAATAATGGAATGTCTTTCAAATCAGAACAG
lytA_dn1000_R	CAATAAATACACGACTAGTCAAACGACAAC
Primers for amplification of ORF of the spectinomycin resistance gene	
Spc_F	GTGAGGAGGATATATTTGAATACATAC
Spc_R	TTATAATTTTTTTAATCTGTTATTTAAATAGTTTATAG
Primers used to confirm gene inactivation, homologous to the inside of the ORF of the spectinomycin resistance gene	
KO vrfy in Spc-1	TCCTGATCCAAACATGTAAGTACC
KO vrfy in Spc-2	AGCAGTTCGTAGTTATCTTGGAGAG

Supplemental Table S2. Primer sequences for RT-PCR

Target		Sequence (5' to 3')
ermB	Forward	AAAACTTACCCGCCATACCA
	Reverse	TTTGGCGTGTTTCATTGCTT
mefA	Forward	CTGTATGGAGCTACCTGTCTGG
	Reverse	CCCAGCTTAGGTATACGTAC
16S rRNA	Forward	TGAGGTAACCGTAAGGAGCCA
	Reverse	TCACCCCAATCATCTATCCCA
